# Supplementary material for: iPSC-derived ITGA6-positive cells restore aqueous humor outflow in glaucoma eyes
Source: Nat Commun. 2025 Oct 27;16:9441. doi: 10.1038/s41467-025-65475-8 (PMC12559184; doi:10.1038/s41467-025-65475-8)
Supplement: Supplementary file 1 — Supplementary Information [file 41467_2025_65475_MOESM1_ESM.pdf]

Supplementary Methods

Human tissues

Eyes from six human donors were obtained at Beijing Tongren Hospital and 1st Affiliated Hospital of Harbin Medical University. Anterior segments of these eyes were used for human TM cell isolation, fluorescence *in situ* hybridization (FISH), and immunohistochemistry (IHC). Eyes from another six human donors were obtained from the Lions Eye Bank (Iowa City, IA) and used for IHC analysis and TM cell culture. Human donor clinical information, including age and gender, are provided (Supplementary Table 1). The protocol for human tissue collection was approved by the Ethics Committee of Beijing Tongren Hospital, the 1st Affiliated Hospital of Harbin Medical University, and the Eye Bank Association of America in accordance with the tenets of the Declaration of Helsinki.

Generation of human induced pluripotent stem cells

hiPSCs were generated through reprogramming of renal urethra epithelial cells collected from two healthy human donors (U1 and U2; Supplementary Table 1). Written informed consent was obtained from the donors or their immediate family, and the reprogramming process was carried out following the guidelines for stem cell clinical research in China (Beijing Cellapybio Biotech Co., Ltd., Beijing, China). Second passage cells were infected with Sendai virus-carrying the Yamanaka factors, Oct4, Sox2, c-Myc, and Klf4 (Cyto TuneTM-iPS 2.0 Sendai Reprogramming Kit; Thermo Fisher, MA) and began to show iPSC morphology after 9 to 28 days. Isolated hiPSCs colonies were seeded in 0.2% Matrigel (Corning, NY) pre-coated plates and maintained in mTeSR-1 medium containing recombinant human basic fibroblast growth factor (rh bFGF) and human transforming growth factor beta (rh TGF-β; STMECELL Technologies, MA). 5 mg/ml collagenase (Sigma-Aldrich, MO) was used to passage the matured hiPSC colonies. hiPSCs at passage 20-30 were tested for the absence of Sendai virus and used for the differentiation study.

Human primary TM (pTM) cell isolation and culture

The TM is visible as a brown ring in the iridocorneal angle and was dissected with a 0.5 mm curette. After rinsing with Dulbecco's modified eagle medium (DMEM; Gibco, Grand Island, NY), the harvested tissue was digested in DMEM containing 4 mg/ml collagenase A (Sigma-Aldrich, St. Louis, MO) and 4 mg/ml human serum albumin (Sigma-Aldrich) at 37 °C for 2 hours. After centrifugation at 350g for 3 minutes, the cell pellets were resuspended in human complete medium comprising 199E medium (Gibco), 20 % fetal bovine serum (FBS; Gibco), 90 μg/ml porcine heparin (Sigma-Aldrich), 20 U/ml endothelial cell growth supplement (Sigma-Aldrich), and 1.7 mM L-glutamine (Sigma-Aldrich), seeded onto 1 % gelatin pre-coated 6-well plates (Thermo Fisher, Waltham, MA) and cultured in an incubator at 5% CO_2 and 37 °C. pTM cells from donor 1 were characterized at passage 2 for expression of TM biomarkers and formation of dexamethasone (Dex) induced cross-linked actin networks (CLANs).

These cells were subsequently used for scRNA-seq. pTM cells of donors 5 to 9 were used to induce differentiation of human induced pluripotent stem cells (hiPSCs).

Cross-linked actin networks (CLANs) formation

TM cells were seeded on poly-D-lysine (Sigma-Aldrich) pre-coated glass coverslips (LIUSHENG, Nantong, China). When reaching a 70% confluency, cells were exposed to 100 nM dexamethasone (DEX; Sigma-Aldrich) for 3 days, fixed in 4 % paraformaldehyde (wt/vol; PFA; Thermo Fisher) and stained with Alexa Fluor^{TM} 488 Phalloidin (Thermo). Cell nuclei were stained with 4′,6-diamidino-2-phenylindole (DAPI; Santa Cruz, Dallas, TX). Dimethyl Sulfoxide (DMSO; Sigma-Aldrich) was used as the vehicle control. CLANs were identified as the polygonal and dome-like arrangement of actin composed of hubs and spokes. Cells forming CLANs were counted from 5 areas in each coverslip and quantified by dividing by the total cell number. Experiments were technically repeated three times by using pTM cells of donor 1.

Preparation of cryosections for IHC analysis

The anterior segments from enucleated human eyes were collected within 6-8 hours of death and dissected in a biosafety hood. Mice eyes were immediately collected after death. After fixation in 4 % PFA (wt/vol; Thermo Fisher), rinsing with DPBS (Thermo Fisher), and infiltration with sucrose solutions at increasing concentrations (13.3 %, 15.0 %, and 16.7 % wt/vol), tissues were embedded in Optimal Cutting Temperature compound (OCT; Sakura, Tokyo, Japan) and cryosectioned on a Leica CM1950 (Leica, Nussloch, Germany). Sections of 10 μm thickness were used for IHC staining.

IHC analysis

Cells growing on poly-D-lysine coated coverslips were fixed with 4% PFA (Thermo Fisher) for 20 minutes. Fixed cells and sections from human donors 1 to 4 were rinsed in Dulbecco's PBS (1 ×; 145 mM NaCl, 8.1 mM $Na_2HPO_4 \cdot 12H_2O$, 1.9 mM $NaH_2PO_4 \cdot 2H_2O$, PH7.2-7.4; DPBS; Thermo Fisher) for 5 mins, and incubated in the blocking solution (DPBS with 1% bovine serum albumin, BSA; Sigma-Aldrich) for 1 hour. The sections were further incubated with the diluted primary antibodies and the corresponding secondary antibodies (Supplementary Table 2). Cell nuclei were stained with DAPI (Santa Cruz). The stained sections were mounted using Neutral Balsam (Solarbio, Beijing, China) and imaged by confocal microscopy (Nikon A1R MP, Tokyo, Japan).

Western blotting (WB)

Proteins were extracted with RIPA buffer containing Halt™ Protease Inhibitor Cocktail (Thermo) and quantified with BCA protein analysis kit (Thermo). 40 μg protein was boiled, separated on a 10% sodium dodecyl sulfate (SDS)-acrylamide gel by electrophoresis and transferred to a polyvinyl difluoride membrane (PVDF; GE Healthcare Life Sciences China, Beijing, China) by Mini Trans-Blot Cell (Bio-Rad) at 150 mA current for 2 hours. The membranes were then incubated in blocking buffer

(Tris-buffered Saline-Tween-20 (TBST) containing 5% non-fat milk powder) at room temperature for 1.5 hours, and incubated with the diluted primary antibodies (Supplementary Table 2) or rabbit polyclonal GAPDH (1:10000; Abcam, ab181602) overnight at 4 °C. Finally membranes were incubated with the secondary antibody conjugated to horseradish peroxidase (HRP; Abcam) at room temperature for 1 hour. Immunoreactive bands were visualized using the enhanced chemiluminescence detection kit (Thermo) and a ChemiDoc XRS+ imaging system (Bio-Rad). Band intensities were quantified using Image Lab software (Bio-Rad) and normalized to GAPDH. Experiments were carried out in triplicate.

Probe preparation for FISH analysis

Total RNA was extracted from TM cells of donor 1 using TRIzol reagent (Sigma-Aldrich). cDNAs were generated from mRNA through a random primed reverse transcription reaction using the FastKing RT Kit (TIANGEN Biotech, Beijing, China). The primers for amplification 350-500 bp probes were designed using Primer3 (http://primer3.ut.ee; Supplementary Table 3). The probes were synthesized by quantitative Polymerase Chain Reaction (qPCR) reaction using digoxigenin-11-dUTP and dNTPs (Roche, Basel, Switzerland), which was run at 94 °C for 5 min, followed by 30 cycles at 94 °C for 30 s, from 55 °C to 45 °C (-0.5 °C per cycle) for 30 s in first 20 cycles and at 45 °C for 30 s in the following 10 cycles, at 72 °C for 30 s. The amplification products were sequenced using a ABI3730XL sequencer (Applied Biosystems, Inc., Carlsbad, California) and those displaying at least 75% homology to the predicted sequence were used as probes (Supplementary Table 3).

FISH analysis

The tissue of donor 1 was fixed in 4% PFA prepared using diethyl pryocarbonate (DEPC; Sigma-Aldrich) treated H_2O for 24 hours, embedded in paraffin, and sectioned on a Leica CM1950 microtome (Leica). Tissue at 4 μm thickness was deparaffinized by 100% xylol (wt/vol; Sinopharm, Shanghai, China) and rehydrated in a series of decreasing ethanol concentration (100 %, 85 %, 70 % wt/vol; Sinopharm). The sections were then incubated in 100% methanol (wt/vol; Sinopharm), Proteinase K solution (1 μg/ml; Sigma-Aldrich), 4% PFA (wt/vol), 1.33% triethanolamine (wt/vol; Sinopharm), 1% Triton-X 100 (wt/vol; Sigma-Aldrich) and pre-hybridization solution (Boster, Wuhan, China) at room temperature for 1 hour. After denaturing for 10 minutes at 80 °C, the probes were incubated with the sections placed in a humid chamber filled with 50% formamide (wt/vol; Sinopharm) for 20 hours at 65 °C. After rinsing with saline sodium citrate buffer (2×, 0.5×, 0.2×SSC; Sigma-Aldrich), the sections were incubated with a blocking solution comprised of 1% bovine serum albumin (wt/vol; BSA; Sigma) and 0.3% Triton X-100 (wt/vol; Sigma-Aldrich) for 1 hour, and incubated with Anti-digoxigenin-fluorescein (1:200; Roche) at 4 °C overnight. Cell nuclei were stained with DAPI (Santa Cruz). The stained sections were mounted using the anti-fading Mounting Medium (S2100, Solarbio) and imaged by confocal microscopy (Nikon, Tokyo, Japan). Experiments were technically repeated three times.

RT-PCR analysis

Total RNA was isolated with TRIzol (Ambion, TX). The concentration and purity of RNA were determined by measuring the absorbance at 260 and 280 nm (A260/280) by Nanodrop (Implen, Germany). cDNAs were generated from RNAs through random primed reverse transcription reaction (Promega, WI). DNA was isolated using TIANamp Genomic DNA Kit (TIANGEN Biotech, Beijing, China, DP304). The target genes were amplified in triplicate using the SYBR Green system (Bio-Rad, Hercules, CA). The primers (Beijing TsingkeBiotechnology Co., Ltd, China) used for amplifying the target genes were listed in Supplementary Table 4, and *GAPDH*/*ACTB* was used as a reference gene. PCR reactions were carried out at 95 °C for 15 min, followed by 50 cycles at 95 °C for 10 s, at 50 °C for 30 s and at 72 °C for 30 s.

Preparation of adenovirus 5 (Ad5)

Mutant MYOC^{Y437H} was created by site directed mutagenesis of T 1309 to C of human myocilin and these constructs were cloned into pBHGlox E1, 3. The plasmids of pDC311 and pBHGlox E1, 3 carrying Myoc^{Y437H} were co-transfected into HEK293 cells to generate the recombinant Ad5 named Ad5-MYOC^{Y437H}-EGFP using LipoFiter^{TM} transfection reagent (Shanghai Hanbio Technology, China). Ad5 virus expressing only EGFP, referred to as Ad5-EGFP, was used as a control. Ad5 stocks containing 3.2×10^{10} plaque formation unit (PFU)/ml were prepared and stored at -80 °C.

Intracameral injections

Mice were subjected to deep anesthesia using 8% chloral hydrate (0.125 ml/20 g). Ad5-MYOCY437H-EGFP (8×10^{7} PFU; 2.5 μl) was manually injected into the anterior chamber of 2-mon-old C57BL/6 mice. Eyes receiving the same amount of Ad5-EGFP were used as controls. In the cell transplantation experiment, 50,000 iPSC-TM cells labeled with PLGA-SPIO-Cypate nanoparticles were resuspended in 3 μl 1×PBS (Gibco) and injected into mouse anterior chamber. Mice having received an equal amount of 1×PBS (Gibco) were used as the control. Eyes were placed adjacent to a ring magnet (inner magnetic field strength of roughly 60 mT from the pupil center of the eye) for 15 minutes. Note that the magnetic field strength was calculated using a Gauss meter (Senjie, Zhejiang, China), and the magnet was oriented parallel to the horizontal plane of the TM region.

Lipofectamine 3000 transfection

NEAT1/*ITGA6* siRNA and scrambled siRNA oligonucleotide (Supplementary Table 5) were prepared (*NEAT1*: Shanghai GenePharma Co., Ltd, China; *ITGA6*: sc-43130, SantaCruz Biotechnology, Inc., Texas, U.S.A) and transfected into iPSC-TM cells using LipoFiter^{TM} transfection reagents (Invitrogen). 48 hours after transfection, cells were collected for RNA extraction and RT-PCR.

NEAT1/*ITGA6* siRNA, scrambled siRNA oligonucleotides, and menRNA (Supplementary Table 5) were prepared (*NEAT1*: Shanghai GenePharma Co., Ltd, China; *ITGA6*: sc-43130, SantaCruz Biotechnology, Inc., Texas, U.S.A; menRNA:

Beijing TsingkeBiotechnology Co., Ltd, China) and transfected into iPSC-TM or pTM cells using LipoFiter^{TM} transfection reagents (Invitrogen). 12 and 48 hours after transfection, cells were collected for RNA extraction and RT-PCR.

Supplementary Figure 1

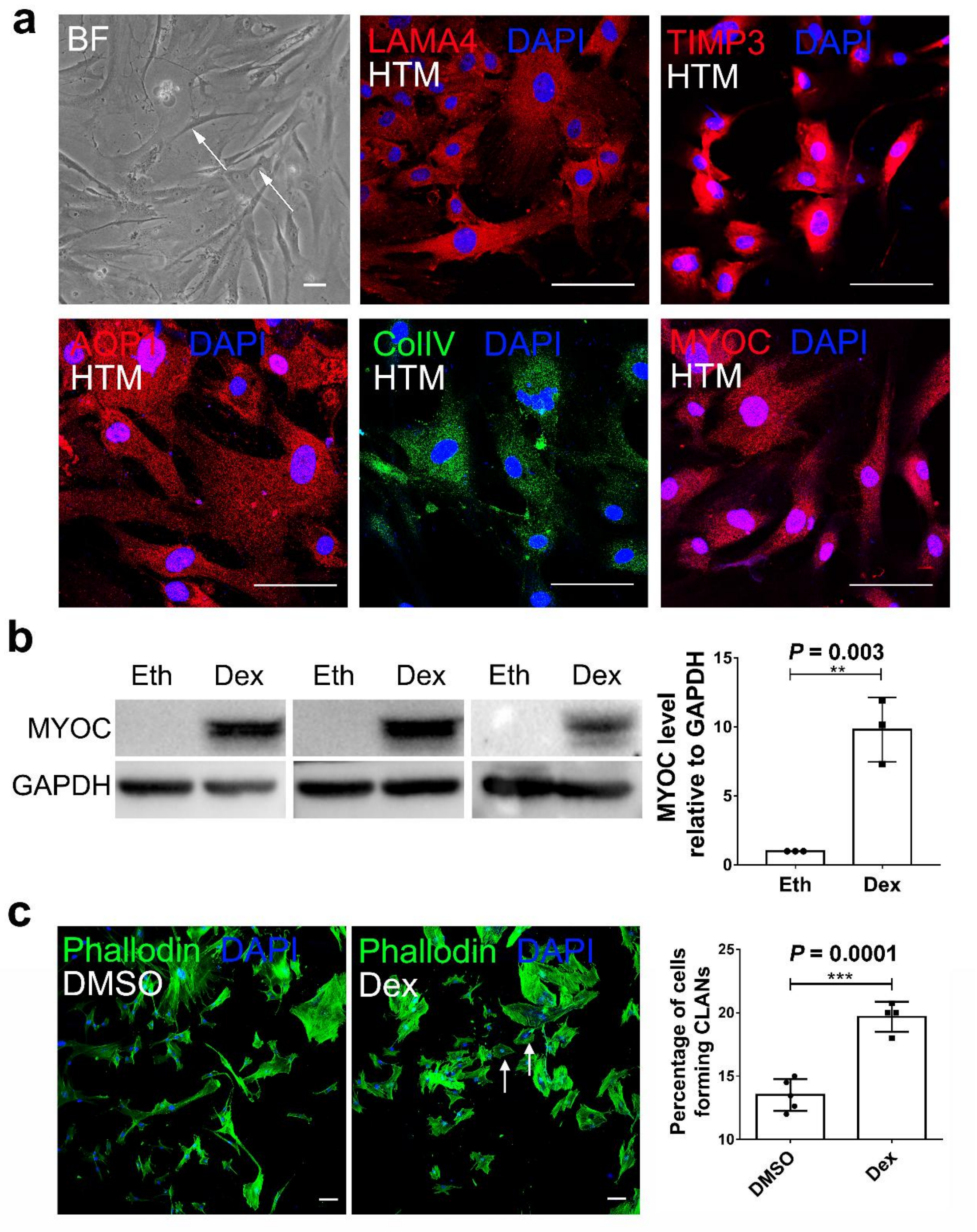

Supplementary Figure 1. Characterization of pTM. (a) A representative image showing pTM morphology with arrows pointing to typical spindle- or cobblestone-shaped cells. Immunohistochemical staining of LAMA4 (red), TIMP3 (red), AQP1 (red), ColIV (green), and MYOC (red) in pTM cells. Nuclei were stained with DAPI

(blue). Experiments were technically repeated three times using pTM cells from donor 1. Scale bars, 50 µm. (b) Left panel: Western blot detection of MYOC (top) or GAPDH (bottom) in pTM of donor 1 after Dex (100 nM) treatment for 3 days. Typical results from n=3 technical repeats are shown with similar results. Right panel: Quantification of band intensities using Image Lab software (Bio-Rad) showing a significantly higher expression of MYOC in pTM after Dex treatment (9.8-fold increase by Dex; Dex: 9.8 ± 2.3 vs. DMSO: 1.0 ± 0.0). (c) Left panel: Immunohistochemical staining of phalloidin (green) in pTM treated with DMSO (n=5 technical repeats) or Dex (100nM; n=4 technical repeats) for 3 days. Nuclei were labeled with DAPI (blue). Scale bars, 100 µm. Right panel: Quantification indicating that Dex treatment leads to a significant increase of CLAN formation in pTM (Dex: 19.7 ± 1.2 % vs. DMSO: 13.5 ± 1.3 %). ***P*<0.01, ****P*<0.001 by two-tailed Student's t-test.

Supplementary Figure 2

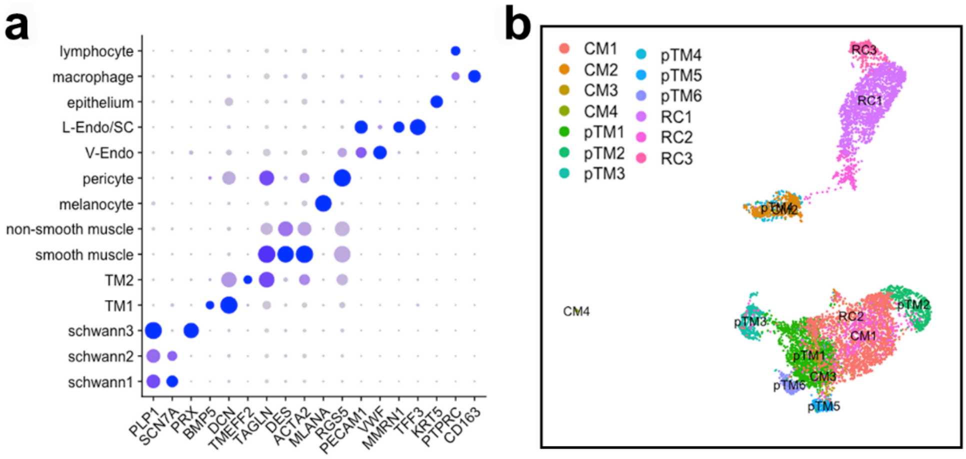

Supplementary Figure 2. Integrated analysis. (a) Dot plot showing the expression of marker genes in the integrated analysis of human outflow tissues. (b) UMAP plot showing the cluster information of pTM, iPSC-TM CM and RC.

Supplementary Figure 3

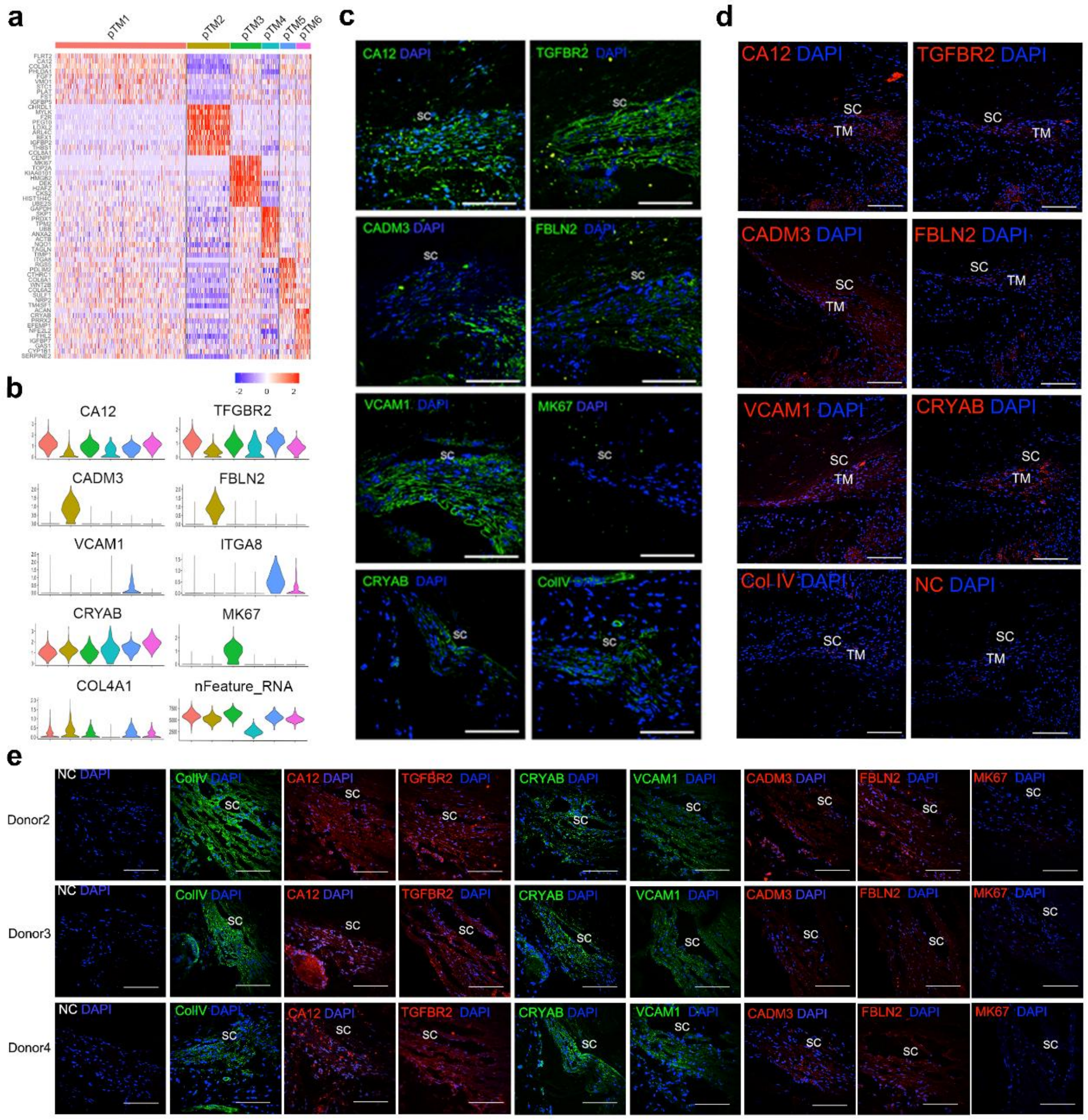

Supplementary Figure 3. Immunohistochemical analysis of DEGs in pTM clusters. (a) Intensities of DEGs in each cluster are displayed as colors ranging from red to blue. Both rows and columns are clustered using correlation distance and average linage. (b) Violin plot showing the expressions of representative DEGs. (c) Immunohistochemical staining of Col IV (green), CA12 (red), TGFBR2 (red), CRYAB (green), VCAM1 (green), CADM3 (red), FBLN2 (red), and MK67 (green) in the tissues of donor 1. (d) FISH analysis using the synthesized probes for *Col IV* (red), *CA12* (red), *TGFBR2* (red), *CRYAB* (red), *VCAM1* (red), *CADM3* (red), and

FBLN2 (red) in the conventional outflow tissue of donor 1. Unstained tissues with the probes were used as the negative controls (NC). (e) Immunohistochemical staining of Col IV (green), CA12 (red), TGFBR2 (red), CRYAB (green), VCAM1 (green), CADM3 (red), FBLN2 (red), and MK67 (green) in human donors 2-4. Unstained tissues with the synthesized probes were used as the negative controls (NCs). Nuclei were labeled with DAPI (blue). Experiments were technically repeated three times. Scale bars, 100 μm. TM: trabecular meshwork; SC: Schlemm's canal.

Supplementary Figure 4

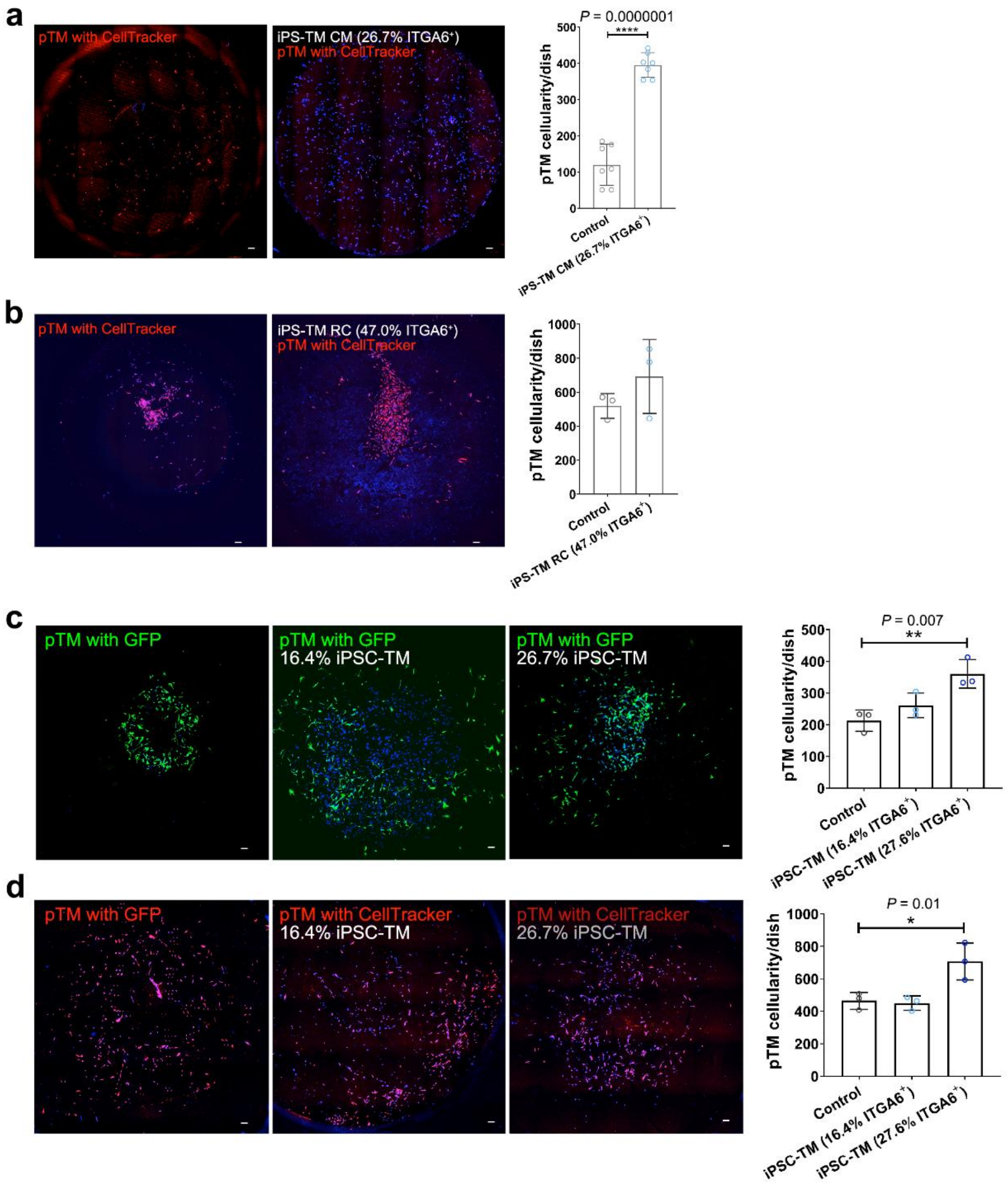

Supplementary Figure 4. Effect of iPSC-TM on pTM proliferation. (a) Left panel: Histochemical staining of pTM cells pre-labeled with CellTracker Red CMTPX after co-culture with iPSC-TM of U2 (CM), where $ITGA6^+$ cell proportion is 26.7%, for 48 hours. Right panel: Quantification of pTM cells (red) showing iPSC-TM of U2 (CM) significantly stimulates pTM division after 48 hours of co-culture (iPSC-TM: 395.3 ± 34.0 vs. pTM: 120.0 ± 56.7). n=7 technical repeats. ****P<0.0001 by two-tailed

Student's t-test. (b). Similar as panel A, showing histochemical staining of pTM (red) after co-culture with iPSC-TM of U2 (RC), where ITGA6$^+$ cell proportion is 47.0%, for 48 hours. Quantification of pTM (red) revealing a similar role of iPSC-TM of U2 (RC) in stimulating cell proliferation (iPSC-TM: 693.0 ± 217.3 vs. pTM: 520.0 ± 72.5). n=3 technical repeats. (c) Left panel: Histochemical staining of pTM cells transfected with lentivirus carrying EGFP after co-culture for 48 hours with iPSC-TM of U1 (CM), where ITGA6$^+$ cell proportions are 16.4% and 27.6%. Right panel: Quantification of pTM cells (green) showing that iPSC-TM of U1 at 27.6% ITGA6$^+$ ratio significantly stimulates pTM division after 48 hours of co-culture (27.6% iPSC-ITGA6$^+$ cells: 361.0 ± 45.1 vs. pTM: 212.0 ± 33.5). n=3 technical repeats. (d) iPSC-TM of U1 (CM) at 27.6% ITGA6$^+$ ratio significantly stimulates pTM division after 48 hours of co-culture using CellTracker Red CMTPX (27.6% iPSC-ITGA6$^+$ cells: 707.3 ± 115.0 vs. pTM: 465.0 ± 51.4). n=3 technical repeats. *P<0.05, **P<0.01 by one-way ANOVA with Tukey's post-hoc test. Nuclei were labeled with DAPI (blue). Experiments were technically repeated three times. Scale bars, 100 µm.

Supplementary Figure 5

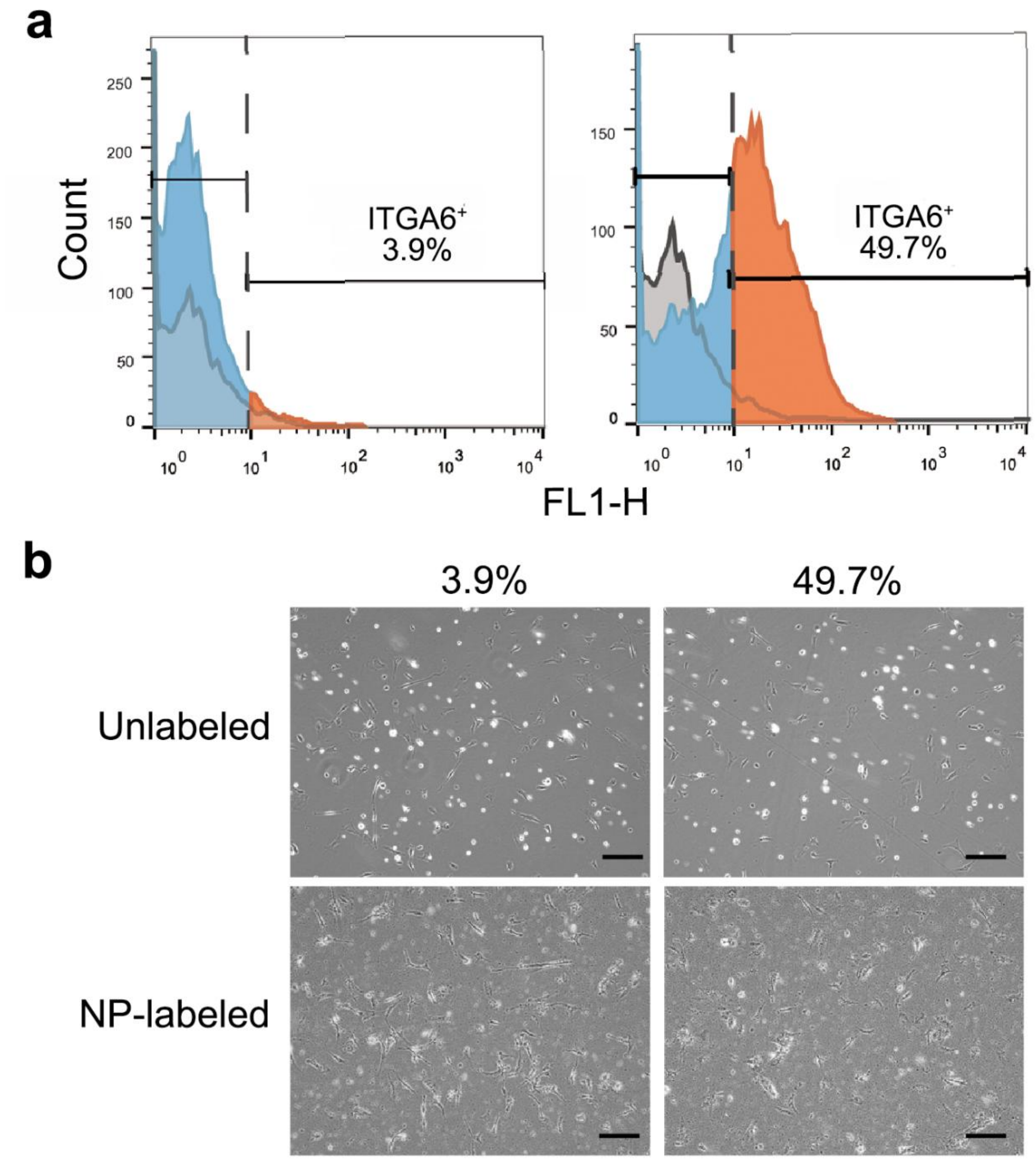

Supplementary Figure 5. Cells transplanted into Tg-MYOC^{Y437H}. (a) Similar as panel A of Figure 3, showing flow cytometry histograms when ITGA6^{+} ratios are 3.9% and 49.7%. (b) Representative images of iPSC-TM morphology before and after purification, as well as after NP-labeling.

Supplementary Figure 6

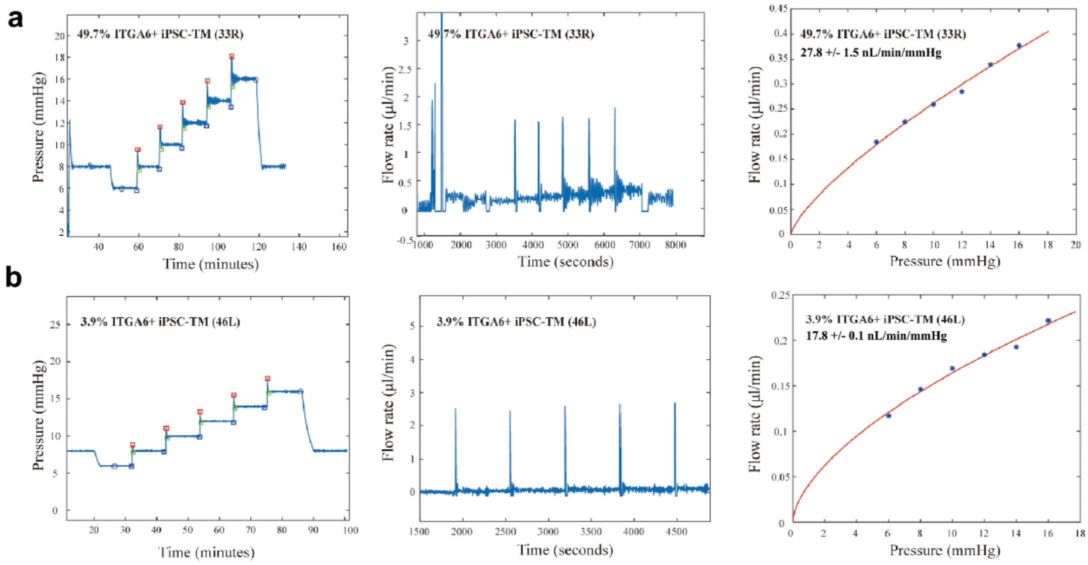

Supplementary Figure 6. Outflow facility measurement to evaluate iPSC-ITGA6$^+$ cell function. Representative pressure-time, flow rate-time, and flow-pressure curves from enucleated Tg-MYOC^{Y437H} eyes treated with 49.7% ITGA6$^+$ iPSC-TM (a) and 3.9% ITGA6$^+$ iPSC-TM (b). Data points are fitted with an existing relationship (power law model).

Supplementary Figure 7

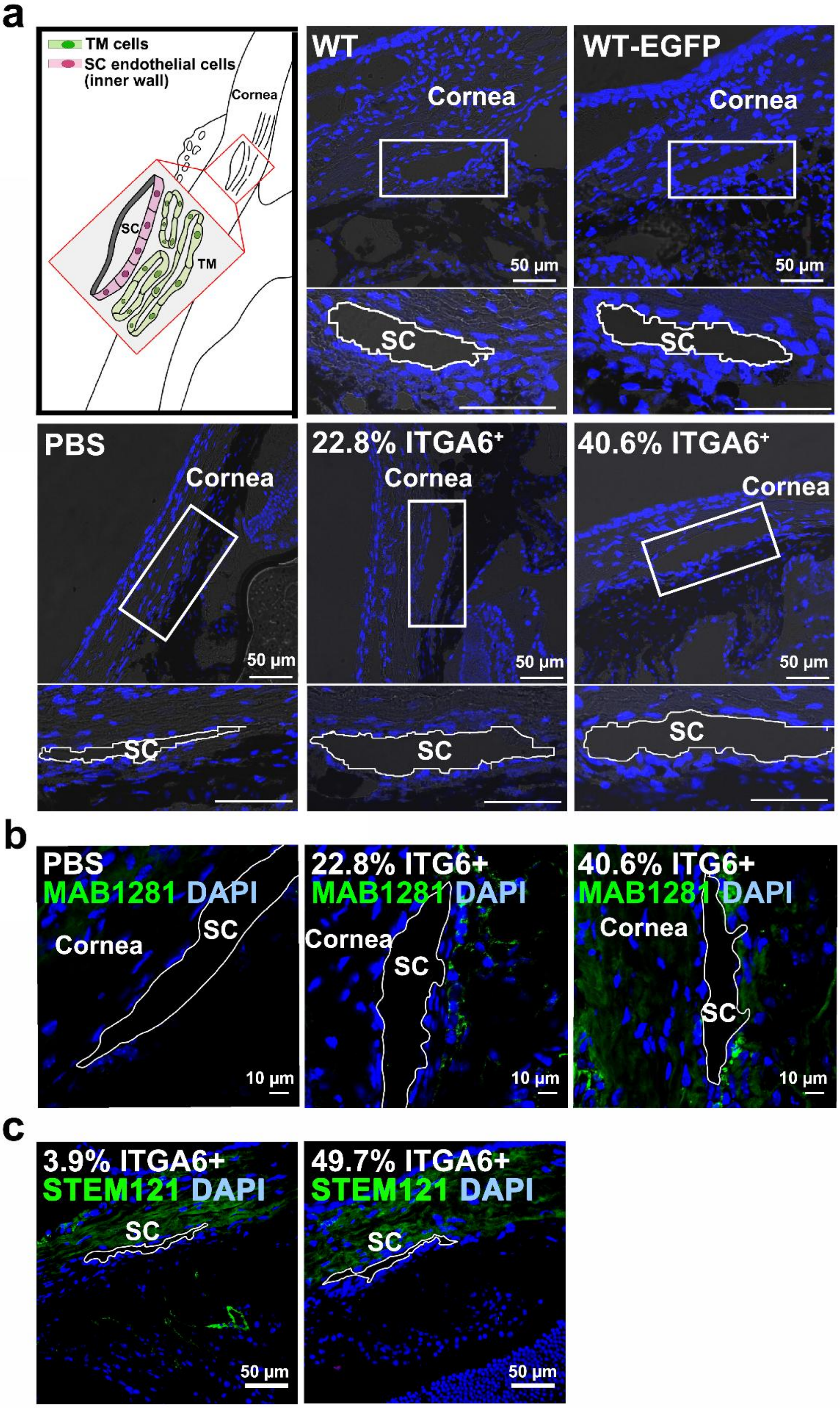

Supplementary Figure 7. IHC analysis of TM and SC after receiving iPSC-ITGA6$^+$ cells. (a) Immunohistochemical staining of nuclei in the iridocorneal regions and a schematic illustration of the anatomical structure of the TM and SC. Higher magnification views are shown at the bottom of each image. Representative results from n=11-28 sections are shown. Scale bars, 50 μm. (b) Immunohistochemical staining of MAB1281 in glaucoma mice treated with iPSC-TM CM containing 22.8% or 40.6% ITGA6$^+$ cells. Typical results from n=10-23 sections are shown. Scale bars, 10 μm. (c) Immunohistochemical staining of nuclei in the iridocorneal regions of Tg-MYOC^{Y437H} mice having received iPSC-TM CM with ITGA6$^+$ contributions of 3.9% and 49.7%. Scale bars, 50 μm.

Supplementary Figure 8

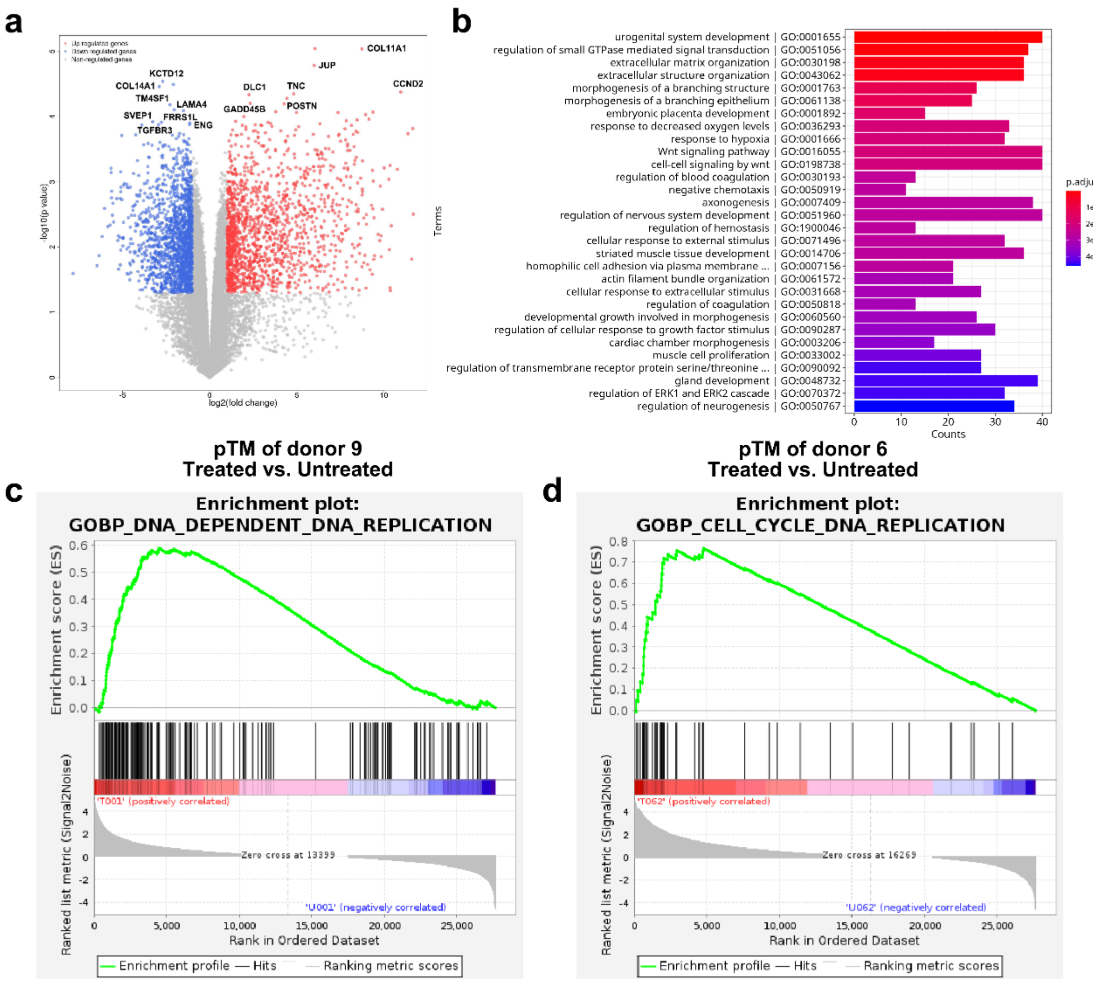

Supplementary Figure 8. Enrichment analysis of DEGs in iPSC-ITGA6^{+} cells and pTM after co-culture. (a) Volcano plot displaying DEGs in pTM of donor 9 after co-culture, associated with cell fate determination and proliferative regulation (Table S8). (b) Biological process enriched in pTM of donor 9 after co-culture, referring to neuron system development and cell proliferation. (c-d) GSEA assay showing DNA dependent DNA replication/cell cycle DNA replication upregulated in pTM of donor 9 (c) and 6 (d) after co-culture.

Supplementary Figure 9

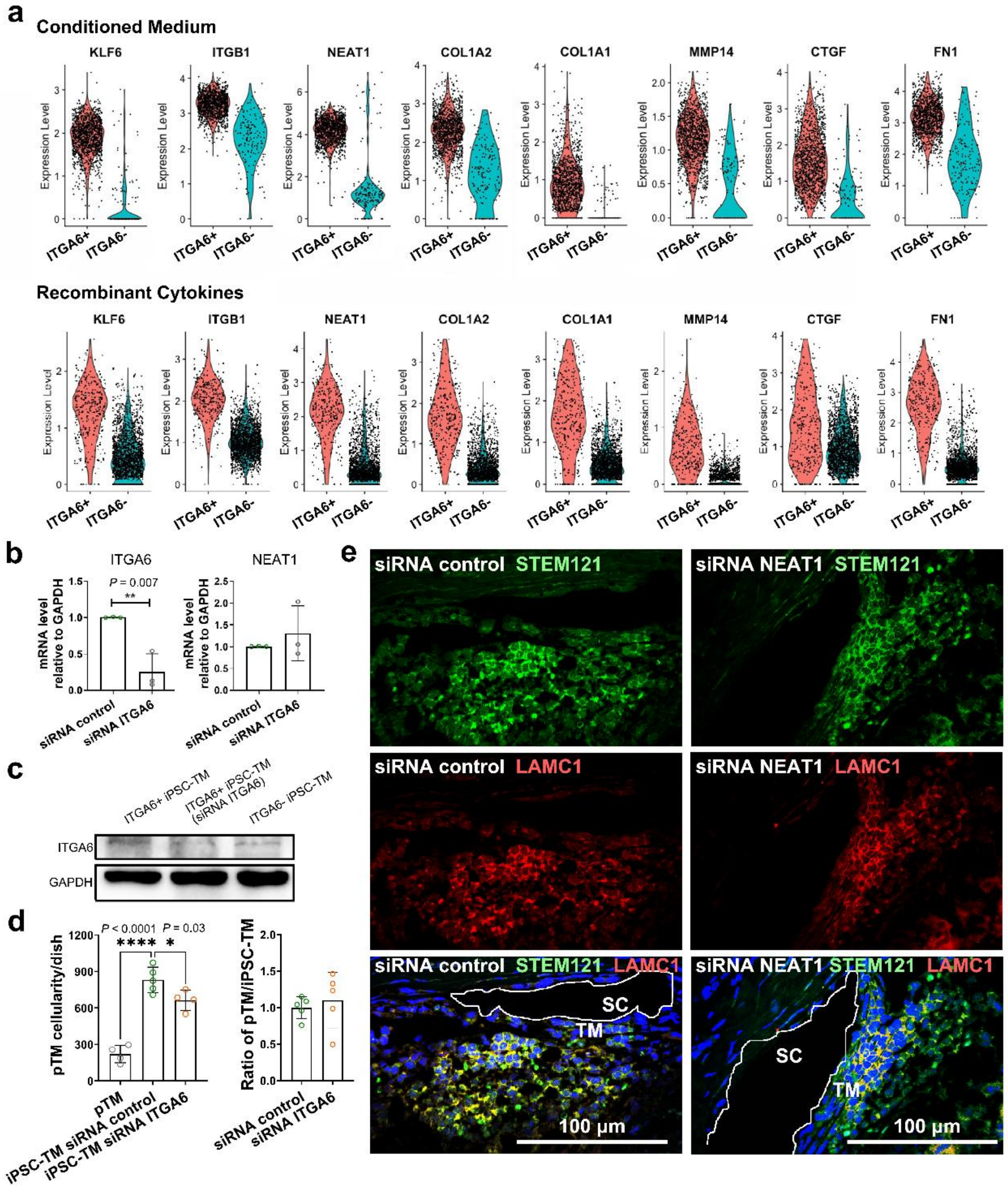

Supplementary Figure 9. Influence of *NEAT1* on iPSC-TM's function. (a) Violin plot showing typical DEGs in ITGA6$^+$ cluster. Violin plot indicating the expressions of *KLF6, ITGB1, NEAT1, Col1A2, Col1A1, MMP14, CTGF,* and *FN1* in ITGA6$^+$ (orange) or ITGA6$^-$ (blue) iPSC-TM CM (up panel)/RC (bottom panel). Each dot indicates a single cell. (b) RT-PCR results showing the expression of *ITGA6* (siRNA control vs. siRNA ITGA6: 1.0 ± 0.006 vs. 0.25 ± 0.25) and *NEAT1* (siRNA control vs.

siRNA ITGA6: 1.0 ± 0.006 vs. 1.3 ± 0.6) in iPSC-TM after treatment with scrambled siRNA (siRNA control) or siRNA ITGA6. Typical results from n=3 are shown. **P<0.01 by two-tailed Student's t-test. (c) Western blot detection of ITGA6 and GAPDH (from top to bottom) in ITGA6$^+$ iPSC-TM, ITGA6$^+$ iPSC-TM transfected with siRNA ITGA6, and ITGA6$^-$ iPSC-TM. The experiment was repeated three times with similar results. (d) Left panel: Quantification of pre-stained pTM cellularity indicating an impaired capacity of iPSC-TM after ITGA6 knockdown in stimulating cell proliferation. In pTM (n=4), and iPSC-TM treated with siRNA control (n=5) and siRNA ITGA6 (n=4), pTM cellularity/dish: 220.3 ± 71.0, 831.8 ± 104.2, and 664.5 ± 84.0. Right panel: A ratio of pTM cellularity compared to iPSC-TM cellularity in each co-culture sample (siRNA ITGA6: 1.1 ± 0.4 vs. siRNA control: 1.0 ± 0.2). Typical results from n=5 are shown. *P<0.05, ****P<0.0001 by one-way ANOVA with Tukey's post-hoc test. (e) Immunohistochemical staining of STEM121 (green) and LAMC1 (red) in the iridocorneal tissues of 2-month-old C57BL/6 mice after receiving 50,000 human iPSC-TM pre-treated with siRNA control or siRNA *NEAT1* for 48 hours. Nuclei were labeled with DAPI (blue). Experiments were technically repeated three times using 57-85 sections of 6 eyes in each group. Scale bars, 100 μm. TM: trabecular meshwork; SC: Schlemm's canal.

Supplementary Figure 10

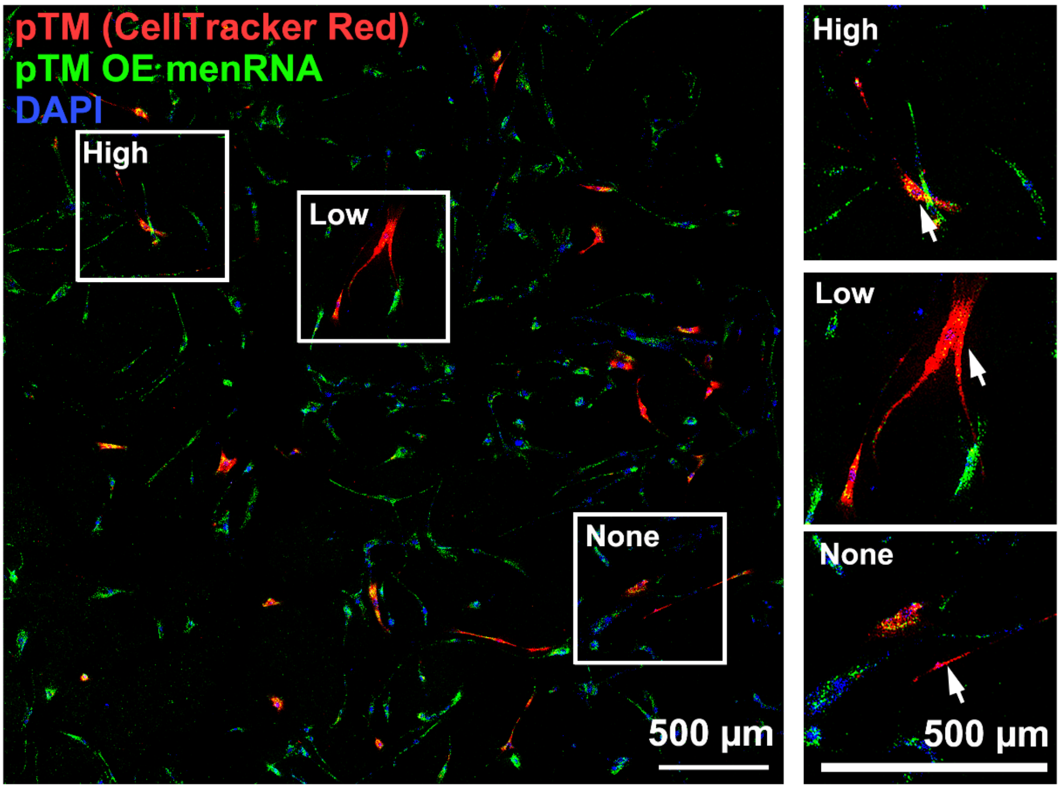

Supplementary Figure 10. menRNA transfer. Representative co-culture images of pTM pre-stained with CellTracker Red and pTM overexpressing menRNA pre-labeled with green fluorescence, indicating different efficiencies of menRNA transfer between cells. High, high efficiency; Low, low efficiency; None, no transfer. Scale bar, 500 μm.

Supplementary Table 1. Human donor demographic characteristics. N/A = not available

Donor	Age	Gender	Eye No.	Application
Donor1	54	Male	18-001	scRNA-seq, Tissue IHC, FISH
Donor2	53	Female	15-009	Tissue IHC
Donor3	66	Female	16-076	Tissue IHC
Donor4	74	Female	17-009	Tissue IHC
Donor5	80	Male	17-056	TM cell culture
Donor6	37	Female	17-062	TM cell culture
Donor7	62	Female	14-010	TM cell culture
Donor8	N/A	N/A	BJ	TM cell culture
Donor9	N/A	Male	23-001	TM cell culture
Donor10	4	Male	23-002	TM cell culture
U1	37	Male	U1	Renal urethra epithelial cells, scRNA-seq, co-culture
U2	28	Female	U2	Renal urethra epithelial cells, co-culture, cell transplantation

Supplementary Table 2. *Information of antibodies for IHC and WB*

**Antibody name**	**Company/Catalog number**	**Dilution ratio/Concentration of working solution**
mouse monoclonal anti-collagen IV(COLIV)	Abcam, ab6311	5 µg/ml
rabbit polyclonal anti-Myocilin antibody	Abcam, ab85842	5 µg/ml
rabbit polyclonal anti-Laminin alpha 4	Abcam, ab209675	1： 200
rabbit polyclonal anti-TIMP3	Abcam, ab39184	3 µg/ml
rabbit monoclonal anti-aquaporin antibody (AQP1)	Abcam, ab168387	1： 500
rabbit polyclonal carbonic anhydrase XII12 (CA12)	Novus, NBP1-81668	1： 500
rabbit polyclonal anti-TGF beta receptor II (TGFBR2)	Abcam, ab78419	1： 200
mouse monoclonal anti-alpha β crystallin (CRYAB)	Abcam, ab13496	1： 100
mouse monoclonal anti-VACM-1	Novus, NBP1-47491	1： 500
rabbit polyclonal anti-CADM3	Novus, NBP1-88604	1： 500
rabbit polyclonal anti-fibulin 2 (FBLN2)	Novus, NBP1-33479	1： 1000
mouse monoclonal ITGA6	Abcam, ab20142	1:500 for IHC
FITC anti-human ITGA6	Miltenyi Biotec, 130-097-245	1:11 for flow cytometry
rabbit monoclonal anti-Integrin alpha 6	Abcam, ab181551	1:2000 for WB
rabbit polyclonal anti-NONO	Novus, NB100-1556	1： 1000
STEM121	TaKaRa, Y40410	1： 500
mouse monoclonal anti-nuclei (MAB1281)	Merck, MAB1281	1： 500
mouse monoclonal anti-SFPQ	Novus, 6D7	1： 2000
rabbit polyclonal anti-Ki-67	Novus, NB500-170	1： 300
rabbit monoclonal recombinant anti-Laminin gamma 1	Abcam, ab233389	1： 2000
anti-mouse IgG Alexa FluorTM 488	Invitrogen, A28175	1： 1000
anti-mouse IgG (H+L) Alexa Fluor® 647	Abcam, ab150115	1： 1000
anti-rabbit IgG Alexa Fluor TM 568	Invitrogen, A11011	1： 500

Supplementary Table 3. Information of probes for FISH analysis

**Name**	**Forward primer (5’-3')**	**Reverse primer (5’-3')**	**Length (bp)**	**Similarity to the target gene (%)**
Col IV	AAGGGTGATACTGGAGAA	CTTGAACTTGAGCTTGTC	483	90.1
CA12	TTTGTGAAGCTGAACCTGCC	CCTGCCGGAAGTTGTTGATC	508	91.2
TGFB2	TCTTGGAAGACAGAGAAGGA	ATGGAGTAGACATCGGTCTG	466	90.7
CRYAB	CGCCTCTTTGACCAGTTCTT	TGACAGCAGGCTTCTCTTCA	418	92.8
ITGA8	GTTACCTTGGATACTCAG	AATCATTCTTGTCTATGTCT	574	93.4
VCAM1	GGGAAAAACAGAAAAGAGGT	CATATCCGTATCCTCCAAAA	445	89.4
CADM3	CATCCCTGCAATGGTCTAAC	TCATGGTTCACAGAGCACAC	443	82.1
FBLN2	CGAGGGCTACCAGTACTATG	CTCCTGGTCATAGCTGTAGG	383	76
MKi67	CTGAAAATAAGGGAATATC	TATGACTTCTGGTTCTTA	474	93..0

Supplementary Table 4. Primers for RT-PCR analysis

Name	Forward primer (5’-3')	Reverse primer (5’-3')
STC1	CGAGTGGCGGCTCAAAACT	AGCAGCGCTGTACAAGAAGGA
NEAT1	TGGCCAAGACAGCCTGTTTC	TCAAATCAACCACCTAAGTTGCTAA
IER3	CCAGCTGCCAGTCGAGGAA	CCAGGGATGCGGCGTTA
MGP	CCTGATCCTTCTTGCCATCCT	AGATTCCATGCTTTCATGTGATTC
AQP1	CCTCCAGCTGGTGCTATGC	AAGGACCGAGCAGGGTTAATC
ABCG2	ATCTTGGCTGTCATGGCTTCA	TCTTCGCCAGTACATGTTGCA
NES	GGCGCACCTCAAGATGTCC	GGGAATTGCAGCTCCAGCT
MKI-67	CAAAGAGTGAGAAAGGCAAAATCA	TGAACTTGCCGACTGCTAGGA
POU5F1	CAGGCCCGAAAGAGAAAGC	CCACACTCGGACCACATCC
SOX2	GAGAACCCCAAGATGCACAAC	TTCTTCATGAGCGTCTTGGTTTT
NOTCH1	CGCCGTGAACAATGTGGAT	GTCCCGGTTGGCAAAGTG
NANOG	TGGGCCTGAAGAAAACTATCCA	GAAGTGGGTTGTTTGCCTTTG
IL1A	TTGTATGTGACTGCCCAAGATGA	TGGATGGGCAACTGATGTGA
SERPINE1	CCACAGACGCGATCTTCGT	TGTGTCTTCACCCAGTCATTGAT
VAV3	GTGTGCCCAAACCAGTAGATTATTCT	TGCTAATTGCATATTCTCCTGACTCT
COL8A1	CGTGTGGGTTGCTCTATTCAAG	GCCTGTTCTGAGGGCATCTG
TYRP1	GACACGCCTCCTTTTTATTCCA	ATGGGTTTGTCCCCCTGTTC
SRY	GAAAATGCTTACTGAAGCCGAA	CAGTTGCACTTCGCTGCAGA
ITGA6	TTCAATATCAATTGTGGGCACACT	TCCATGCACACTTTCTGTTTCTG
NEAT1_2	TGACCCCTCCACGTGTACACT	AAGCAGAAAGAGGACCCTGGAT
menRNA	ATGATGGGAAGTGACATGCGT	TGGGCCTTCGTACTCTTCTCT
FOXC1	CGGCTTGAACAACTCTCCAG	CTTTTCCTGCTTTGGGGTTCG
ZFP42	CTCACAGTCCAGCAGGTGTT	CATGTTTTCCTGCCTGTGCC
TBX3	AAGTCGGGAAGGCGAATGT	GCCACCATCCACCGAGAAT
TFAP2C	TGCCTATGTCTGTGAAGCCG	TTTACACAGTTGCTGGGCCG
DUSP6	CTGGTGGTGGCACGTC	CTGCGGGGACTCGAACC
ACTB	GTGCCCATCTACGAGGGGTAT	GTAGCACAGCTTCTCCTTAATGTCA
GAPDH	CATGTTCGTCATGGGTGTGAA	GGCATGGACTGTGGTCATGAG

Supplementary Table 5. Sequences of Scrambled siRNA, *NEAT1 siRNAs, and ITGA6 siRNA.*

Name	Sequences
Scrambled siRNA	UUCUCCGAACGUGUCACGUTT
NEAT1siRNA-1	GCAGGUUGAAGGGAAUUCUTT
NEAT1 siRNA-2	CAGGAGGCUACCAUUUAAATT
NEAT1 siRNA-3	GGUGUUAUCAAGUGAAUUATT
ITGA6 siRNA-1	CCAUCACAGUAACUCCUAAtt
ITGA6 siRNA-2	GGAUAUGCCUCCAGGUUAAtt
ITGA6 siRNA-3	CCAAACUGAUCCAGUAUAAtt
menRNA	GGCGCUGGUGGUGGCACGUCCAGCACGGCUGG GCCGGGGUUCGAGUCCCCGCAGUGUUG
