# Supplementary material for: iPSC-derived ITGA6-positive cells restore aqueous humor outflow in glaucoma eyes
Source: Nat Commun. 2025 Oct 27;16:9441. doi: 10.1038/s41467-025-65475-8 (PMC12559184; doi:10.1038/s41467-025-65475-8)
Supplement: Supplementary file 2 — Description of Additional Supplementary Files [file 41467_2025_65475_MOESM2_ESM.pdf]

Title: Supplementary Data 1

Description: Interactions of integrins and their ligands

Title: Supplementary Data 2

Description: Networks of pTM or iPSC-TM and major cell types in human conventional outflow tissues

Title: Supplementary Data 3

Description: DEGs in pTM of donors 6 and 9 after co-culture

Title: Supplementary Data 4

Description: DF and F in key findings after multiple comparisons

Title: Supplementary Data 5

Description: FPKM and fold changes in Figures 4, 6, and 8
