# Supplementary material for: iPSC-derived ITGA6-positive cells restore aqueous humor outflow in glaucoma eyes
Source: Nat Commun. 2025 Oct 27;16:9441. doi: 10.1038/s41467-025-65475-8 (PMC12559184; doi:10.1038/s41467-025-65475-8)
Supplement: Supplementary file 8 — Reporting Summary [file 41467_2025_65475_MOESM8_ESM.pdf]

nature portfolio

Corresponding author(s): Wei Zhu
Ningli Wang
Wenhua Xu

Last updated by author(s): Sep 27, 2025

Reporting Summary

Nature Portfolio wishes to improve the reproducibility of the work that we publish. This form provides structure for consistency and transparency in reporting. For further information on Nature Portfolio policies, see our Editorial Policies and the Editorial Policy Checklist.

Statistics

For all statistical analyses, confirm that the following items are present in the figure legend, table legend, main text, or Methods section.

n/a	Confirmed	
☐	☒	The exact sample size (*n*) for each experimental group/condition, given as a discrete number and unit of measurement
☐	☒	A statement on whether measurements were taken from distinct samples or whether the same sample was measured repeatedly
☐	☒	The statistical test(s) used AND whether they are one- or two-sided<br>*Only common tests should be described solely by name; describe more complex techniques in the Methods section.*
☐	☒	A description of all covariates tested
☐	☒	A description of any assumptions or corrections, such as tests of normality and adjustment for multiple comparisons
☐	☒	A full description of the statistical parameters including central tendency (e.g. means) or other basic estimates (e.g. regression coefficient) AND variation (e.g. standard deviation) or associated estimates of uncertainty (e.g. confidence intervals)
☐	☒	For null hypothesis testing, the test statistic (e.g. *F*, *t*, *r*) with confidence intervals, effect sizes, degrees of freedom and *P* value noted<br>*Give P values as exact values whenever suitable.*
☐	☒	For Bayesian analysis, information on the choice of priors and Markov chain Monte Carlo settings
☐	☒	For hierarchical and complex designs, identification of the appropriate level for tests and full reporting of outcomes
☐	☒	Estimates of effect sizes (e.g. Cohen's *d*, Pearson's *r*), indicating how they were calculated

Our web collection on statistics for biologists contains articles on many of the points above.

Software and code

Policy information about availability of computer code

Data collection	Nikon A1R MP HD and Zeiss LSM Zen were applied for imaging. Fiji and CountStar was applied for cell counting. BD FACSCalibur CellQuest and FlowJo 10 were used in flow cytometry. Homolab was applied for the collection of flow rates and pressures in Syringe-pump system. Gen5 was used to analyze the protein concentration. Gel Imaging System of Bio-Rad was applied in the Western blot. Nanodrop was applied for RNA concentration measurement. QuantStudioTMDesign & Analasis was applied for the data collection of RT-PCR. Cell Ranger, Seurat, Canonical Correlation Analysis, CellPhoneDB, ClusterProfiler, FastQC, Cluster 3.0, KOBAS 2.0 and GSEA were used for the data analysis of scRNA-seq and bulk RNA-seq.
Data analysis	For two-group comparison, two-tailed Student's t-test, Mann-Whitney test, and normality and lognormality test were used for statistical analysis of CLANs formation, RT-PCR results, outflow facility data, ITGA6 intensity, and paraspeckle amount. For multiple group comparisons, one-way ANOVA with Tukey's post-hoc test was performed for statistical analysis of the cellularity data, and two-way ANOVA with Tukey's post-hoc test was applied for the statistical analysis of IOP results. ANOVA tables, including the degree of freedom and F-values, were provided in Supplementary Data 4. All tests were performed in GraphPad Prism 7. Data are expressed as the mean ± SD. P values < 0.05 were considered to be statistically significant.

For manuscripts utilizing custom algorithms or software that are central to the research but not yet described in published literature, software must be made available to editors and reviewers. We strongly encourage code deposition in a community repository (e.g. GitHub). See the Nature Portfolio guidelines for submitting code & software for further information.

Data

Policy information about availability of data

All manuscripts must include a data availability statement. This statement should provide the following information, where applicable:

- Accession codes, unique identifiers, or web links for publicly available datasets
- A description of any restrictions on data availability
- For clinical datasets or third party data, please ensure that the statement adheres to our policy

Raw data files have been uploaded in the National Library of Medicine of the National Center for Biotechnology Information with BioProject ID: PRJNA948656 (scRNA-seq) and PRJNA1098785 (RNA-seq). Source data are provided with this paper.

Research involving human participants, their data, or biological material

Policy information about studies with human participants or human data. See also policy information about sex, gender (identity/presentation), and sexual orientation and race, ethnicity and racism.

Reporting on sex and gender	This study includes 6 females, 5 males, and one donor for whom information is missing. Sex and gender were determined based on self-reporting, which were not considered in study design.
Reporting on race, ethnicity, or other socially relevant groupings	This study includes 6 Chinese donors and 6 Caucasian donors. Samples from these donors were used for TM cell collection, tissue dissection, and iPSC generation. Race was not considered in study design.
Population characteristics	The samples were grouped randomly, and each examination involved at least three donors.
Recruitment	Lions Eye Bank (Iowa City, IA), Beijing Tongren Hospital (Beijing, China), and 1st Affiliated Hospital of Harbin Medical University (Harbin, China)
Ethics oversight	The protocol for human tissue/cell collection was approved by the Ethics Committee of Beijing Tongren Hospital, the 1st Affiliated Hospital of Harbin Medical University, and the Eye Bank Association of America in accordance with the tenets of the Declaration of Helsinki.

Note that full information on the approval of the study protocol must also be provided in the manuscript.

Field-specific reporting

Please select the one below that is the best fit for your research. If you are not sure, read the appropriate sections before making your selection.

☒ Life sciences ☐ Behavioural & social sciences ☐ Ecological, evolutionary & environmental sciences

For a reference copy of the document with all sections, see nature.com/documents/nr-reporting-summary-flat.pdf

Life sciences study design

All studies must disclose on these points even when the disclosure is negative.

Sample size	Power analysis (https://www.gigacalculator.com/calculators/power-sample-size-calculator.php) was performed to determine the sample size in our animal study. We chose the Type I error rate to be 0.05 and power to be 0.8.
Data exclusions	IOP measurements were taken between 9:00 and 12:00 am, and data represent the average of three-five measurements. If 1) tonometer cannot read the pressure; 2) three-five measurements are variable (> 3 mmHg), measurements were excluded.
Replication	Experiments were repeated at least three times.
Randomization	Samples/animals were allocated to experimental groups and processed randomly.
Blinding	Experiments occurred in a double-blinded fashion.

Reporting for specific materials, systems and methods

We require information from authors about some types of materials, experimental systems and methods used in many studies. Here, indicate whether each material, system or method listed is relevant to your study. If you are not sure if a list item applies to your research, read the appropriate section before selecting a response.

Materials & experimental systems

n/a	Involved in the study
	x Antibodies
	x Eukaryotic cell lines
x	Palaeontology and archaeology
	x Animals and other organisms
x	Clinical data
x	Dual use research of concern
x	Plants

Methods

n/a	Involved in the study
x	ChIP-seq
	x Flow cytometry
x	MRI-based neuroimaging

Antibodies

Antibodies used

mouse monoclonal anti-collagen IV(COLIV) Abcam, ab6311 5 μg/ml
rabbit polyclonal anti-Myocilin antibody Abcam, ab85842 5 μg/ml
rabbit polyclonal anti-Laminin alpha 4 (LAMA4) Abcam, ab209675 1:200
rabbit polyclonal anti-TIMP3 Abcam, ab39184 3 μg/ml
rabbit monoclonal anti-aquaporin antibody (AQP) Abcam, ab168387 1:500
rabbit polyclonal carbonic anhydrase XII12 (CA12) Novus, NBP1-81668 1:500
rabbit polyclonal anti-TGF beta receptor II (TGFBR2) Abcam, ab78419 1:200
mouse monoclonal anti-alpha β crystallin (CRYAB) Abcam, ab13496 1:100
mouse monoclonal anti-VACM-1 Novus, NBP1-47491 1:500
rabbit polyclonal anti-CADM3 Novus, NBP1-88604 1:500
rabbit polyclonal anti-fibulin 2 (FBLN2) Novus, NBP1-33479 1:1000
mouse monoclonal ITGA6 Abcam, ab20142 1:500 for IHC
FITC anti-human ITGA6 Miltenyi Biotec,
130-097-245 1:11 for flow cytometry
rabbit monoclonal anti-Integrin alpha 6 Abcam, ab181551 1:2000 for WB
rabbit polyclonal anti-NONO Novus, NB100-1556 1:1000
STEM121 TaKaRa, Y40410 1:500
mouse monoclonal anti-nuclei (MAB1281) Merck, MAB1281 1:500
mouse monoclonal anti-SFPQ Novus, 6D7 1:2000
rabbit polyclonal anti-Ki-67 Novus, NB500-170 1:300
rabbit monoclonal recombinant anti-Laminin gamma 1 Abcam, ab233389 1:2000
RFP (dsRed) monoclonal antibody Invitrogen, MA5-15257 1:100
anti-mouse IgG Alexa FluorTM 488 Invitrogen, A28175 1:1000
anti-mouse IgG (H+L) Alexa Fluor® 647 Abcam, ab150115 1:1000
anti-rabbit IgG Alexa Fluor TM 568 Invitrogen, A11011 1:500

Validation

Anti-Collagen IV antibody, Reacts with Human; Suitable for IHC-P;
Anti-Myocilin antibody, Reacts with Human, Cynomolgus monkey; Suitable for ELISA and IHC-P;
Anti-Laminin alpha 4 antibody, Reacts with Human; Suitable for ICC-IF, IHC-P and WB;
Anti-TIMP3 antibody, Reacts with Mouse, Human; Suitable for ICC-IF and WB;
Anti-Aquaporin 1 antibody, Reacts with Mouse, Rat, Human; Suitable for WB, IHC-P, Flow Cyt, ICC-IF;
Anti-Carbonic anhydrase XII antibody, Reacts with Human; Suitable for WB, ICC-IF, IHC, IHC-P;
Anti-TGF beta receptor II antibody, Reacts with Human; Suitable for Flow Cyt, IHC-P;
Anti-Alpha B crystallin antibody, Reacts with Mouse, Rat, Chicken, Cow, Human; Suitable for WB, ICC, IP, IHC-IF, Flow Cyt, IHC-P;
Anti-VCAM-1 antibody, Reacts with Human, Mouse; Suitable for WB, ELISA, IB, IHC, IHC-P, IP;
Anti-CADM3 antibody, Reacts with Human, Mouse, Rat; Suitable for WB, IHC, IHC-P;
Anti-Fibulin 2 antibody, Reacts with Human, Mouse, Rat; Suitable for WB, IHC, IHC-P;
Anti-Integrin alpha 6 antibody, Reacts with Human; Suitable for Flow cyt, ICC-IF, IHC, IHC-P;
Anti-NONO antibody, Reacts with Human, Mouse, Rat, Orangutan; Suitable for WB, IHC, IHC-P, IP, ICC-IF;
Anti-STEM121 antibody, Reacts with Human; Suitable for IHC, IF;
Anti-SFPQ, Reacts with Human; Suitable for WB, ELISA, ICC/IF, IHC;
Anti-MKI67 antibody, Reacts with Human, Mouse, Rat, Porcine, Avian; Suitable for WB, Flow Cyt, IB, ICC-IF, IHC-Fr, IHC-P, IP;
Anti-MAB1281 antibody, Reacts with Human only, Suitable IHC, IP, ICC;
Anti-laminin gamma1, Reacts with: Mouse, Rat, Human; Suitable for: WB, IHC-P, IP, Flow Cyt;
Anti-RFP antibody, Reacts with RFP from the Discosoma sea anemone N-terminal peptide-KLH conjugated; Suitable for WB, IHC, ICC-IF, ELISA, IP;
Anti-GAPDH antibody, Reacts with Mouse, Rat, Chicken, Human, Zebrafish, African green monkey, Xenopus tropicalis; Suitable for Flow Cyt, WB, IHC-P, ICC-IF, IP

WB: Western Blot;
IHC: Immunohistochemistry;
IHC-P: Immunohistochemistry-Paraffin;
ICC: Immunocytochemistry;

IF: Immunofluorescence;
IP: Immunoprecipitation;
Flow Cyt: Flow Cytometry;
ELISA: Enzyme linked immunosorbent assay;
IB: Immunoblotting.
IHC-Fr: Immunohistochemistry-Frozen

Eukaryotic cell lines

Policy information about cell lines and Sex and Gender in Research

Cell line source(s)	Donor Age Gender Race Cause of Death Ophthalmic disease Eye No. Application<br>Donor 1 54 Male Chinese Encephalorrhagia N 18-001 scRNA-seq, Tissue IHC, FISH<br>Donor5 80 Male Caucasian Acute respiratory distress N 17-056 TM cell culture<br>Donor6 37 Female Caucasian Acute liver failure N 17-062 TM cell culture<br>Donor7 62 Female Caucasian Breast cancer N 14-010 TM cell culture<br>Donor8 N/A N/A Chinese N/A N BJ TM cell culture<br>Donor 9 N/A Male Chinese N/A N 23-001 TM cell culture<br>Donor 10 N/A Male Chinese Lung cancer N 23-002 TM cell culture<br>U1 37 Male Chinese A N U1 Renal urethra epithelial cells, scRNA-seq, co-culture<br>U2 28 Female Chinese A N U2 Renal urethra epithelial cells, co-culture, cell transplantation
Authentication	The protocol for human cell collection was approved by the Ethics Committee of Beijing Tongren Hospital, the 1st Affiliated Hospital of Harbin Medical University, and the Eye Bank Association of America in accordance with the tenets of the Declaration of Helsinki.
Mycoplasma contamination	All cell lines were tested negative for mycoplasma contamination using Quick Cell Mycoplasma Test Kit (C4056L1060, LIFE iLAB Bio Co., Ltd, Shanghai, China).
Commonly misidentified lines (See ICLAC register)	No misidentified cell lines was used in this study.

Animals and other research organisms

Policy information about studies involving animals; ARRIVE guidelines recommended for reporting animal research, and Sex and Gender in Research

Laboratory animals	C57BL/6 mice were purchased from Beijing Vital River Laboratory Animal Technology Co., Ltd. (Beijing, China). Transgenic mice expressing human myocilinY437H (Tg-MYOCY437H) were a kind gift of Professor Val C. Sheffield (University of Iowa). Tg-MYOCY437H mice with elevated IOPs were selected for cell therapy.
Wild animals	No wild animals was used.
Reporting on sex	Sex was not considered in study design.
Field-collected samples	Mice were housed under standard condition with a 12 hour/12 hour day/night cycle, at 23 ± 2 °C temperature and 50 ± 5% humidity.
Ethics oversight	All experiments were conducted according to the ARVO Statement for the Use of Animals in Ophthalmic and Vision Research and the laboratory animal care and use guidelines of Qingdao University Medical Center and Beijing Tongren Hospital.

Note that full information on the approval of the study protocol must also be provided in the manuscript.

Plants

Seed stocks	n/a
Novel plant genotypes	n/a
Authentication	n/a

Flow Cytometry

Plots

Confirm that:

- [x] The axis labels state the marker and fluorochrome used (e.g. CD4-FITC).
- [x] The axis scales are clearly visible. Include numbers along axes only for bottom left plot of group (a 'group' is an analysis of identical markers).
- [x] All plots are contour plots with outliers or pseudocolor plots.
- [x] A numerical value for number of cells or percentage (with statistics) is provided.

Methodology

Sample preparation	To determine the ratio of ITGA6+ cells in the samples, 30,000 purified cells were rinsed with 1×PBS (Gibco) and suspended in 1×PBS buffer containing 1 % FBS (Gibco). After incubation with FITC anti-human ITGA6 antibody (Miltenyi Biotec; 1:11) for 30 minutes at 4 °C, cells were rinsed and resuspended with 500 µl buffer for flow cytometry analysis.
Instrument	BD FACSCalibur (Becton Dickinson)
Software	FlowJo
Cell population abundance	ITGA6+ cells were purified from a cell mixture using a magnetic-activated cell sorting (MACS) system, and cell population abundance was determined by flow cytometry analysis. Untreated cells were used as negative controls. Based on negative controls, 10^1 FL1-H was determined as the threshold for fluorescence-positive cells. This standard has been followed throughout the entire study. The ratio of fluorescence-positive cells was analyzed by BD FACSCalibur (Becton Dickinson, NJ, USA).
Gating strategy	The Voltage and Amp Gain of the Forward scatter (FSC) was E00 and 1.00, that of the side scatter (SSC) was 340 and 1.00. The voltage of fluorescence2 (FL1) was 381. In the FSC/SSC plot, we aimed to collect all living cells in the sample. In the FL1-H plot, 10^1 FL1-H was determined as the threshold for fluorescence-positive cells, as described earlier.

- [x] Tick this box to confirm that a figure exemplifying the gating strategy is provided in the Supplementary Information.
